# Risk of Cancer for Workers Exposed to Antimony Compounds: A Systematic Review

**DOI:** 10.3390/ijerph16224474

**Published:** 2019-11-14

**Authors:** Anton Saerens, Manosij Ghosh, Jelle Verdonck, Lode Godderis

**Affiliations:** 1 Department of Public Health and Primary Care, Centre Environment & Health, 3000 Leuven, Belgium; anton.saerens@student.kuleuven.be (A.S.); manosij.ghosh@kuleuven.be (M.G.); jelle.verdonck@kuleuven.be (J.V.); 2 External Service for Prevention and Protection at Work, Idewe, 3001 Heverlee, Belgium

**Keywords:** antimony, cancer, occupational health, systematic review, risk assessment

## Abstract

Background: Antimony (Sb) trioxide and antimony trisulfide are “2B: Possibly carcinogenic to humans” and “3: Unclassifiable” according to the International Agency for Research on Cancer (IARC). The U.S. National Toxicology Program (NTP) concluded that antimony trioxide “is reasonably anticipated to be a human carcinogen based on studies in rats and mice”. We investigated the cancer hazard of antimony compounds for workers, a population with high exposure to antimony substances. Methods: Using the “Guidelines for performing systematic reviews in the development of toxicity factors” (Texas Commission on Environmental Quality (TCEQ) 2017) as a guidance, we established a human and an animal toxicology data stream in Medline and ToxLine. Data from this review were applied in a human health risk assessment. Results: A final pool of 10 occupational and 13 animal toxicology articles resulted after application of TCEQ guidelines. Conclusions: Antimony carcinogenicity evidence involving workers is inadequate, based on confounding, small sample sizes, incomparability across studies, and inadequate reference populations. An increased lung cancer risk cannot be excluded. Evidence for lung neoplasms caused by antimony trioxide inhalation in experimental animals is sufficient. Overall, carcinogenicity in workers is probable (International Agency for Research on Cancer (IARC) 2A). It remains unclear from what occupational exposure duration and dose this effect arises and whether exposure threshold values should be reconsidered.

## 1. Introduction

### 1.1. Chemical Properties

Antimony (Sb) is a chemical which resides in the group of semimetals. It may naturally appear in oxidation states of −III, 0, +III, or +V with Sb (III) being more stable than Sb (V). The electroneutral form is the white elemental crystalline Sb. In stibine (SbH_3_), it is trivalent negative. In its positive trivalent state, Sb may exist as antimony trioxide (Sb_2_O_3_), antimony trisulfide (stibnite, Sb_2_S_3_), antimony potassium tartrate (APT), antimony trifluoride, antimony triiodide, antimony acetate, amongst others. In its positive pentavalent form, it may be a part of antimony pentafluoride or antimony pentachloride [1]. It is thought that the trivalent form is more active in vivo [2]. Prevalent antimony compounds are listed in Table 1.

### 1.2. Sources of Exposure

Antimony is naturally present in soils and released to the environment predominantly from anthropogenic sources in industrial processes. As such, it is present in the atmosphere in very low levels. Workers are a population with particularly high exposure to antimony species. Processes involving antimony mostly include metal mining, smelting, and refining activities. Other sources of environmental release are the production, use, and disposal of Sb alloys and compounds, the production of polyethylene terephthalate (PET), soda glass, and fire retardants, the incineration of waste, fuel combustion, and lastly, shooting activities [1,3,4]. In these industries, workers are exposed to multiple antimony and non-antimony species, of which some are (potentially) carcinogenic (e.g., arsenic). Consequently, many studies are not of speciation and often consider jointly other concomitant (potentially) carcinogenic substances. 

### 1.3. Routes of Exposure

Several routes of exposure to antimonials exist. Patients suffering from parasitic diseases like schistosomiasis or leishmaniasis may be treated intravenously with antihelminthic pentavalent antimonials such as antimony potassium tartrate (APT). Environmental exposure routes are through inhalation, oral ingestion by contamination, or hand-to-mouth contact (drinking water, beverages, and food) [5] and dermal contact [1,2,6,7]. This environmental exposure always is a combination of several antimony compounds. The reason for this is twofold. Firstly, in the atmosphere, the supposedly most predominant form of antimony is antimony trioxide (and other oxides to a lesser extent)[8], whereas when dissolved in aqueous media, antimony is largely in the +V oxidation state [1]. Secondly, interconversion is possible in the environment [9] as well as in vivo (see Toxicokinetics section below). As a consequence, studies on antimony exposure in workers have focused on antimony in general, and not on specific valence states nor species of antimony [9].

### 1.4. Toxicokinetics

Data on toxicokinetics of antimony are scarce and research predominantly involves pentavalent antimonials in parenteral treatment of parasitic diseases. After systemic absorption, antimony is distributed to the erythrocytes, liver, kidney, spleen, bone, lung, and thyroid tissue. Sb(+V) can be reduced to Sb(+III) and conjugated with glutathione and to a higher degree, Sb(+III) is oxidized into Sb(+V) [10,11]. Trivalent antimony is excreted mainly through feces via biliary ways, whereas pentavalent antimony is mainly excreted in urine [1]. Half-lives depend on the specific antimony compound as well as route of exposure. In human lung tissue, a very long biological half-life (more than 600 days) is assumed, based on a study with accidental exposure to Sb-125 [10]. When administered intramuscularly, a 95% excretion in urine within 6 h was reported for antimony (V) stibogluconate [11]. 

### 1.5. Health Effects

Health effects of antimony were described in 1571 by Severin as “vomare, cacare, sudare” [12]. Today, these effects still apply, as exposure to antimony may cause gastrointestinal symptoms like nausea, vomiting, anorexia, abdominal pain, and stomach ulcers. Transient irritating skin rashes called antimony spots arise when workers are exposed to antimony in hot weather. Furthermore, antimony has been related to respiratory disease (pneumoconiosis, pleural adhesions, rhinitis, bronchitis, and respiratory irritation with chronic cough), cardiovascular changes (increased blood pressure, electrocardiographic (ECG) changes), and reproductive effects (menstrual abnormalities and spontaneous abortions) although evidence for these effects is limited [8,12]. 

### 1.6. Evidence Concerning Carcinogenicity

The body of evidence concerning the possible carcinogenic effects of antimony and its compounds is limited. This is contrary to arsenic, a similar semimetal chemical [13] that has been established as a carcinogen. One of the reasons for this could be the relatively low use of antimonials in industrial applications [14]. In 1989, the International Agency for Research on Cancer (IARC) published the Monograph on antimony trioxide and antimony trisulfide. Sb_2_O_3_ was classified as possibly carcinogenic to humans (Group 2B), Sb_2_S_3_ was not classifiable as to its carcinogenicity to humans (Group 3) [15]. In 2016, the US’s National Toxicology Program (NTP) finalized its animal toxicology studies on inhalation exposure to antimony trioxide, concluding that antimony trioxide causes lung neoplasms in both sexes of mice and rats [16]. Subsequently, this organization published its Draft Report on Carcinogens Monograph on antimony trioxide, concluding that “antimony (III) trioxide is reasonably anticipated to be a human carcinogen based on sufficient evidence of carcinogenicity from studies in experimental animals and supporting data from mechanistic studies” [9]. In 2017, the Agency for Toxic Substances and Disease Registry (ATSDR) republished their Toxicological Profile for antimony and compounds [6]. This report does not state a final evaluation on the carcinogenicity of antimony compounds. Summarized, the research of NTP has raised new concern about the carcinogenic effects of antimony compounds, especially antimony trioxide. 

### 1.7. Exposure Thresholds

Industrial exposure thresholds have been formulated. For inhalation, the American Conference of Governmental Industrial Hygienists (ACGIH) put the threshold limit value (TLV) for an eight-hour time weighted average (TWA) on 0.5 mg/m^3^ (antimony compounds in general, including antimony trioxide) and 0.1 mg/m^3^ (stibine) [17]. The National Institute for Occupational Safety and Health- recommended exposure limit (NIOSH REL) is TWA on 0.5 mg/m^3^ and Occupational Safety and Health Administration- permissible exposure limits (OSHA PEL) is TWA on 0.5 mg/m^3^. Antimony trioxide is a chemical under study by ACGIH and may move forward with a Notice of Intended Change (NIC) proposal in 2019.

### 1.8. Study Rationale

The recent NTP Report on Carcinogens Monograph on Antimony Trioxide [9] found that there is sufficient evidence of the carcinogenicity of antimony trioxide in experimental animals. This finding raised a new concern as to potential carcinogenic effects in humans. Therefore, we decided to conduct a systematic review focusing on workers, a population with the highest exposure to these chemicals and therefore at the highest odds for cancer. The goal of this study is twofold. The main objective is the identification of a potential cancer hazard for exposed workers through a systematic literature review. The secondary objective, if such a hazard exists, is a human health risk assessment. This assessment will examine whether this hazard poses a threat to occupational health in real-life situations. 

We consider the evidence for inhalation, oral ingestion, and dermal contact, examining occupational cohorts as well as animal studies. As far as we know, this is the first systematic review assessing cancer risk specifically in workers for all possible exposure routes.

## 2. Materials and Methods 

As a guidance, we used the Texas Commission on Environmental Quality (TCEQ) “Guidelines for performing systematic reviews in the development of toxicity factors”, as described by Schaefer and Myers [18]. 

### 2.1. Overview of Study Design 

An overview of our study design can be found in Table 2. We first defined PECO (Population, Exposure, Comparison, and Outcome), followed by a literature search and selection of occupational and animal studies. Articles were then critically appraised (risk of bias assessment) and evidence in occupational and animal settings separately integrated. After taking into account mechanistic elements, suggestions for further research were formulated. Eventually all of the previous elements were integrated into a general conclusion. 

### 2.2. PECO

We first defined the research question through PECO as shown in Table 3. In this case, the population consisted of workers or occupationally exposed people, the exposures were all possible antimony compounds through all exposure routes possible in workers (as mentioned above). Comparison groups were comparable reference populations with no exposure or exposure beneath a threshold producing cancer effects and the outcomes were premalignant or cancerous lesions.

### 2.3. Data Streams

Two separate data streams were established, the first being the human (occupational) toxicology stream and the second being the animal toxicology stream. We did not perform an extensive mechanistic search, given the research question primarily involves workers. Nevertheless, a summary of the mechanistic evidence is given in the discussion.

### 2.4. Literature Search

Based on PECO, we developed search strings for Medline (National Center of Biotechnology Information, US National Library of Medicine, 8600 Rockville Pike, Bethesda MD, USA; website: https://www.ncbi.nlm.nih.gov/pubmed/) and Toxline (National Center of Biotechnology Information, US National Library of Medicine, 8600 Rockville Pike, Bethesda MD, USA; website: https://toxnet.nlm.nih.gov/cgi-bin/sis/htmlgen?TOXLINE) databases. We chose to use separate search strings for human toxicology studies as opposed to animal toxicology studies, the human studies being specifically aimed at occupation and the animal studies aimed at rodents. We started with the development of the search string for Medline and subsequently adapted key words to better fit in ToxLine. As certain relevant studies on animal toxicology, especially genotoxicity and bone marrow toxicity, were left out by the initial search string, we decided to execute an additional search with new search terms in Medline and ToxLine. Supplementary Table S1 shows the development of such search strings. We finally used the search terms described below.

### 2.5. Human Toxicology Studies

In PubMed, we used the search terms (“Occupational Exposure” [Mesh] OR “work*” [All Fields]) AND (“antimony” used the search terms (“Occupational Exposure” [Mesh] OR “work*” [All Fields]) AND (“antimony” [Mesh] OR “antimony” [All Fields]) AND (“Neoplasms” [Mesh] OR “cancer” [All Fields] OR “canc*” [All Fields]) on 28 June 2018 and this search yielded 26 hits. In ToxLine, we used the corresponding search terms (occupational exposure OR work*) AND antimony AND (cancer OR neoplas*) on 28 June 2018 and this search yielded 113 hits. 

### 2.6. Animal and Mechanistic Toxicology Studies

In PubMed, we first used the search terms (“antimony” [Mesh] OR “antimony” [All Fields]) AND (“Neoplas*” OR “cancer” [All Fields] OR “carcinog*” [All Fields]) NOT (“sentinel” OR leishman* OR schistosom*). This search yielded 117 hits on 28 June 2018. In ToxLine, we first used the search terms antimony AND (Neoplas* OR cancer OR carcinog*) on 03 July 2018 and this yielded 392 search hits.

An additional PubMed (on 28 June 2018) and a ToxLine (on 03 July 2018) search was executed, with the following search terms, for PubMed and ToxLine respectively: (“antimony” [MeSH Terms] OR “antimony” [All Fields]) AND (“toxicity” [Subheading] OR “toxicity” [All Fields]) AND (“*In Vivo*” [Journal] OR “*In Vivo* (Brooklyn)” [Journal] OR (“in” [All Fields] AND “vivo”[All Fields]) OR “*in vivo*” [All Fields]), yielding 45 hits; “antimony” AND “toxicity” AND “*In Vivo*” yielding 31 hits.

### 2.7. Inclusion/Exclusion Criteria

We developed inclusion/exclusion criteria, based on PECO and the Schaefer and Myers [18] guidelines. These can be found in Supplementary Material Table S2. For some of these criteria, we considered an explication of their rationale to be warranted.

- Studies on poisoning and overdose were excluded, because it is likely that these effects would require a much higher dose than potential carcinogenic doses;
- Studies not involving mammalian species were excluded because of their doubtful applicability to humans;
- Studies in which exposure to antimony compounds is established through unnatural routes, such as intramuscular (IM)/subcutaneaous (SC)/ and intraperitoneal (IP) injection have questionable relevance;
- Studies on anticarcinogenic effects in cancer cells were excluded as one single molecule may enhance malignant transformation of healthy cells as well as inhibit the proliferation and growth of tumor cells [19], for instance consider the possibility that a certain compound induces mutations and at the same time inhibits angiogenesis.

### 2.8. Study Selection

Two reviewers, M.G. and A.S. independently reviewed the search results, based on title, abstract, full text, inclusion criteria, and exclusion criteria. Disagreements between both reviewers were discussed until final consensus and duplicate articles were removed to arrive at a final data set for human studies, animal studies, and mechanistic studies, respectively. If no full text could be obtained, we based ourselves on the abstract [20]. In one case, more data was obtained through the NTP Monograph on antimony trioxide [21]. Supplementary Material Tables S3 and S4 list the study selection.

#### 2.8.1. Human Toxicology Studies

The reviewers agreed to include 7 articles from the PubMed search and 11 articles from the ToxLine search. Regarding PubMed articles, there was no discussion between both. Regarding ToxLine articles, A.S. did not include Sweeney 1985 [22] and Wingren 1990 [23], as antimony is not mentioned in the abstract of those articles. After reading the full text, both agreed to include these 2 articles additionally. As Gerhardsson 1988 [24] used the same data as Gerhardsson 1993 [25], we only reviewed the latter. A pooling of remaining articles from both search engines resulted in 10 articles with full-text access.

#### 2.8.2. Animal Toxicology Studies 

The reviewers agreed to include 2 articles from the first PubMed search and 10 articles from the first ToxLine search. Regarding PubMed articles, there was a discussion about 4 more articles (Zhang 2018 [26], Kotsopoulos 2012 [27], Rossi 1987 [28], and Sunderman 1983 [29]). All 4 were eventually excluded based on the exclusion criteria: Zhang 2018 [26] was no pure animal study because it studied human prostate cancer cells (LnCaP) implanted in mice, Kotsopoulos 2012 [27] was no animal study, Rossi 1987 [28] had uncertain relevance to health endpoint, and Sunderman 1983 [29] met exclusion criterion intramuscular administration. Regarding ToxLine articles, 3 more articles were discussed (Zhang 2018 [26], Rossi 1987 [28], and Sunderman 1983 [29]), which had been excluded before. 

As Dieter 1991 [30] used the same data as Dieter 1992 [31], we only reviewed the latter. The reviewers agreed to include 5 articles from the additional PubMed search and 7 articles from the additional ToxLine search. Zhang 2018 [26] had already been excluded after discussion of other search results. Gurnani 1993 [32] was excluded upon reading the full text as it included the same data [9,33] as Gurnani 1992 (1) [34].

Full text of 2 articles was not available: Watt 1983 [21] and Gurnani 1992 (2) [20]. Data of the first article could be found in the NTP Monograph on antimony trioxide, whereas for the second one, we were limited to the abstract. Pooling of remaining articles from all animal searches resulted in 13 articles. 

### 2.9. Data Extraction

Data was subsequently extracted. In the occupational studies, we characterized the following wherever possible: industry/occupation, sample size and characteristics, study type, time window in which exposure took place, dose of exposure, duration of exposure and principal findings. It was not possible for any of the studies to establish a no observed adverse effects level (NOAEL) nor to establish the lowest observed adverse effects levels (LOAEL). In animal studies, we characterized the following wherever possible: Antimony compound, study animal, sample size, route of exposure, dose of exposure, duration of exposure, principal findings, and LOAEL/NOAEL where possible.

### 2.10. Critical Appraisal: Study Quality and Risk of Bias (ROB) Assessment

Studies included in the individual data streams were assessed according to the TCEQ’s guidance score criteria. (See Tables 5–8 in Schaefer and Myers, 2017 [18].) In this paper, we mentioned the most important drawbacks of all studies included. Subsequently, we tried to label studies as key, supportive, or informative in nature.

### 2.11. Evidence Integration

Afterwards, evidence was integrated in the separate data streams and finally integrated across data streams.

## 3. Results

### 3.1. Data Extraction 

#### 3.1.1. Human Toxicology Studies

Data of individual studies can be found in Table 4. Only two of the human toxicology studies specified the antimony species to which workers were exposed. In Jones 1994 [35], these were antimony oxides, in Gerhardsson 1993 [25], sulfides and oxides were among the exposures. Most studies [25,35,36,37] were performed in metal smelting and refinery processes. Three studies by the same author examined glassworks [4,5,23]. For other industrial processes, one study could be found for the following: Antimony smelting [38], fur manufacturing [22], and steel manufacturing [39]. The study types were predominantly retrospective occupational cohorts [4,5,23,35,38,39,40]. Gerhardsson 1982 [37] and 1993 [25] were case-control studies.

#### 3.1.2. Animal Toxicology Studies

Data of individual studies can be found in Table 5. The substances most studied are antimony trioxide in inhalation [16,21,41,42], ingestion [33,34,42,43], and dermal contact studies [16] as well as antimony potassium tartrate, only in oral ingestion via food or water [31,44,45,46]. Antimony ore concentrate (principally trisulfide, also called stibnite in less amounts, as well as oxides such as valentinite, senarmontite, and cervantite) was examined in one inhalation study [41], as was antimony trichloride [20]. Exposed animals were mice, rats, and voles. Exposure doses varied widely and exposure duration ranged from single gift to two years to lifelong exposures of more than 2.5 years.

### 3.2. Critical Appraisal: Study Quality and Risk of Bias (ROB) Assessment

#### 3.2.1. Human Toxicology Studies

Supplementary Table S5 displays the critical appraisal according to the TCEQ’s guidance score criteria. 

Major weaknesses hampering the interpretation and validity of findings are common across multiple studies. These problems are evaluated now. Afterwards, the final selection of informative (I), supportive (S), and key (K) studies is based on this appraisal. First and probably most importantly, in all studies antimony was one of many exposures. Some of these exposures (arsenic, cobalt, asbestos, smoking, and polycyclic aromatic hydrocarbons (PAHs)) are known causes of cancer. For instance, Wingren et al. [4] and Jones et al. [36] showed significant correlations between exposure to antimony, lead, and arsenic. This makes attributing risk to individual compounds difficult as confounding effects are very likely. None of the studies could sort out the individual effects of metals, nor by stratification nor by multiple regression analysis [4]. 

Second, sample sizes were rather small [25]. Given the probable smaller effect sizes of exposure to antimony, it is doubtful that any effect could have been found with those samples.

Third, results of human research papers included in this review cannot be reliably compared. Multiple reasons exist:

- Individual studies involved different industries (metal mining vs. metal refinery vs. glass production);
- Exposure dose and duration measurements of antimony and other (potential carcinogenic) substances are incomplete. There were three studies [35,38,39] in which antimony concentrations in the air were sampled.

Fourth, many studies examined multiple organs and multiple substances. This multiple testing may have caused findings based purely on chance. In two studies [4,5] this possibility is even stronger, given that results with *p* < 10% were considered to be significant.

Fifth, it is unclear whether latency times for cancer manifestation were sufficiently accounted for. Three studies suggest a latency time of at least 20 years [35,38,39]. Related to this, it is worth noting that most studies are mortality studies, in which findings are based on mortality causes. This could have led to an underestimation of neoplasms, as workers may have died with cancer and not because of it.

Sixth, a similarity of reference populations compared to the worker populations can be questioned. This is illustrated by Schnorr 1995 [38]. When the reference population for a standardize mortality ratio (SMR) calculation was ethnic specific (based on Hispanic surnames), significant differences in cancer mortality were seen. Those differences disappeared when compared to US white populations.

Overall, the studies least suffering from the drawbacks mentioned above are Jones 1994 [35], Schnorr 1995 [38], and Jones 2007 [36]. With the risk of bias tool, they consequently scored the highest. Of these three, we considered Schnorr 1995 [38] the study with the highest validity and relevance. It was most specific in that it studied antimony workers (as opposed to most other human toxicology studies), confounding exposures (smoking, arsenic) were more or less ruled out, and exposure doses were more or less known. Limited confidence in comparison groups is the major drawback of that paper. Therefore, this study could not justify a key (K) rating and thus it was attributed a supportive/key (S/K) rating. Jones 1994 (major confounding) and Jones 2007 (use of weighing model) received a supportive rating. All other human studies were considered informative. 

#### 3.2.2. Animal Toxicology Studies

Supplementary Table S6 displays the critical appraisal according to the TCEQ’s guidance score criteria. More recent studies were attributed a higher confidence rating, which is based on methodological progress. Critical appraisal of individual studies is discussed below. Afterwards, the final selection of informative (I), supportive (S), and key (K) studies is based on this appraisal. As carcinogenic effects have a long latency time, studies with long exposure and observation periods were deemed more relevant in this selection. 

##### Antimony Trioxide

In the Watt [21] paper, one of the major concerns is its potential confounding. The use of large exposure chamber with pigs inside and pine shavings increased the chance of exposure to non-Sb_2_O_3_ particles [47]. Other major concerns are that only females were studied, necropsy was not complete, randomization was not reported, and exposure levels may have been different from those reported [9,47]. Compared to other antimony trioxide studies, the sample size was rather small. Thus, this study is rated informative (I).

The research by Groth [41] should be interpreted cautiously, given that there was merely one exposure level which was only consistently reached after five months [9,41]. Moreover, exposure was impure (to about 20%), although exposure to known carcinogens (arsenic, lead) was not considered sufficient to induce carcinogenesis. Randomization was questioned in the NTP Monograph [9]. Overall, this study is considered informative (I).

The study by Ainsworth [42] did not explicitly assess cancer development. Moreover, exposure duration was short (12–60 days), sample sizes were rather small, and results are not clearly reported. As such, this study is informative (I) to this review.

In the Gurnani [34] study, only male mice were examined in the subchronic (21 days) exposure group. The clastogenicity studied is an intermediate endpoint to cancer development. The health of animals is questioned, as well as the purity and amount [47] of exposure, given they died at the maximum dose of 1000 mg/kg body weight, which is considered non-lethal [9,33]. No positive control was used, as is now standard practice in clastogenicity studies [33]. Therefore, this study is rated informative (I).

The research by Newton [47] did not report significance. It was however calculated afterwards by NTP [9]. Exposure levels were relatively low and did not reach the maximal tolerated dose. Being methodologically sound, this study is deemed supportive (S).

The study by Elliott [43] had a short exposure duration (single gift or 21 days). Only male animals were studied in the liver DNA repair assay. Outcomes are not immediately relevant to reference value development. Thus, we consider this study informative (I).

The study by Kirkland [33] is regarded as informative (I) as it did merely study micronucleated polychromatic erythrocytes (MNPCE) and chromosomal aberrations (CA), not cancer or preneoplastic lesions. Moreover, this research had a small sample size and a short exposure.

We consider the animal research by NTP [16] key (K) in this review. It got the highest ROB score. Methodologically, it is robust, peer reviewed, and described in profound detail. Potential for confounding is low.

##### Antimony Trichloride

A critical appraisal of the Gurnani 1992 (2) [20] study was not possible based on the abstract. As a consequence, this study is considered informative (I).

##### Antimony Potassium Tartrate (APT)

Studies on APT suffer from several drawbacks. The methods of Kanisawa 1969 [44] are inaccurate as there was no fixed exposure duration, only one exposure group was used, the histopathologic assessment of cancer development is not described in detail, and the actual exposure dose was not measured [31] and might have been higher than foreseen. Furthermore, a high spontaneous tumor rate was observed. Schroeder 1970 [45] suffers from similar methodological drawbacks [31]. Moreover, their sample was struck by a pneumonia epidemic causing premature death. Exposure to APT was toxic given it caused shorter survival in treated animals. Methods of Dieter 1992 [31] were more accurate however, exposure groups were rather small (five per sex per exposure group) as was time window of exposure (14 days, this was an orientational study before execution of a more extensive intraperitoneal exposure study), which is not considered in this review. Poon 1998 [46] had solid methods however there was a relatively short exposure duration of 90 days.

Overall, we can say that chronic exposure studies of APT suffer from methodological drawbacks and that methodologically sounds studies are hampered by relatively short exposure durations. Given these limitations, we labeled the three oldest studies [30,44,45] as informative (I) and the Poon 1998 [46] study as supportive (S).

##### Antimony Ore Concentrate (mostly Stibnite, Sb_2_S_3_)

Although the Groth [41] study had large exposure groups and long exposure duration, findings of this paper should be interpreted cautiously, given that there was only one exposure level which was only consistently reached after five months [9,41]. Moreover, exposure was impure (to about 50%), although exposure to known carcinogens (arsenic, lead) was not considered sufficient to induce carcinogenesis. Randomization was questioned in the NTP Monograph [9]. Overall, this study is considered informative (I).

## 4. Discussion

### 4.1. Evidence Integration

#### 4.1.1. Carcinogenicity in Workers

##### Inhalation, ingestion, and dermal contact

Evidence for inhalation, ingestion, and dermal contact was considered simultaneously since individual studies did not mention the exact exposure route. In the studies rated highest (key/supportive and supportive), an increased lung cancer mortality was a recurrent finding. Schnorr [38] found a relative risk of 1.39 (*p* < 0.1) related to exposure duration. Jones [35] found a relative lung cancer risk of 1.55 (*p* = 0.016) for workers recruited before 1961. Jones [36] found a significant correlation (*p* < 0.05) between lung cancer risk and antimony exposure in a weighted model of three exposure scenarios. Among the studies deemed informative, some studies (Sweeney [22], Wingren [5,23], and Finkelstein [39]) found an increase in lung cancer as well. Hepatobiliary cancer mortality was increased in the key/supportive study by Schnorr [38] with a SMR of 3.17 (*p* < 0.05) and 1.58 (*p* > 0.05), depending on the reference population. This excess was observed as well in the informative study by Finkelstein [39]. Other cancers were only increased in one or more informative studies, colon [4,5,23], pharynx [23]. For stomach cancer, Wingren [4,5] saw contradictory effects.

##### Summary

Overall, an increased lung cancer risk in exposed workers is possible from multiple studies, (Grade C, Evidence Based Medicine (EBM) level 2B). Risk evaluation for other cancer types was not possible based on the available studies.

#### 4.1.2. Carcinogenicity in Animals

##### Inhalation 

In the NTP [16] key study, antimony trioxide induced lung adenoma, lung carcinoma, and lung lesions which are considered preneoplastic (e.g., alveolar/bronchiolar epithelium hyperplasia) in male and female mice. In rats, no increase in lung carcinoma was observed. An increase in lung adenoma was seen in females. Lesions considered preneoplastic (e.g., alveolar/bronchiolar hyperplasia) were seen in both sexes. In the Newton [47] supportive study, exposed rats showed lung lesions considered preneoplastic by NTP (e.g., alveolar/bronchiolar epithelium hyperplasia, inflammation) but no increase in adenoma or carcinoma. In the Watt [21] and Groth [41] informative studies, lung carcinomas were increased in female rats.

Squamous metaplasia of the epiglottis was seen in mice with subacute exposure to antimony trioxide at higher exposure doses. No other study found similar effects. Furthermore, in the NTP key study, antimony trioxide induced pheochromocytoma (benign in male, benign/malignant in female) and adrenal medulla hyperplasia in rats. No other study found similar effects. An increase in lymphoma in female mice was observed in the same study. No other study found similar effects. In the informative study by Groth [41], antimony ore concentrate induced lung neoplasms in female rats.

##### Ingestion

The studies [33,34,42,43] considering antimony trioxide were all considered informative. No major adverse effects were observed. Elliott noted a decrease in polychromatic erythrocytes (PCE), which could suggest bone marrow toxicity [43].

One informative study involved antimony trichloride. This study concluded that the substance induces chromosomal aberrations in bone marrow cells [20]. For antimony potassium tartrate, we considered the study by Poon [46] supportive. In this study, dose dependent histologic changes were seen in thyroid, liver, spleen and thymus. The authors saw non-dose dependent changes in the pituitary gland. Concerning the informative papers, Kanisawa [44] saw no adverse effects, Schroeder [45] saw an increase in mortality, and Dieter [31] saw a decrease in weight gain, hepatocellular centrilobular cytoplasmic vacuolization, stomach inflammation, necrosis, ulceration, and squamous epithelium hyperplasia in mice whereas they saw no adverse effects in rats. 

##### Dermal contact

The NTP key studies of mice [16] observed significant differences upon exposure to antimony trioxide in skin histology with a significant increase of benign fibrous histiocytoma and fibrous histiocytoma/fibrosarcoma in males and a non-significant increase in squamous cell metaplasia in females.

##### Summary

Overall, an increased risk of lung cancer from inhalation of antimony species (especially antimony trioxide) in animals is evident from the articles reviewed (Grade A, EBM 1a levels). This conclusion is based on an observed increase of lung cancer cases and preneoplastic lung lesions in exposed animals in a key study, reinforced by supportive and informative studies. The species for which this was made clear is antimony trioxide. There is some evidence of neoplastic or preneoplastic effects of antimony species in animals in other tissues and organs (epiglottis, adrenal medulla, lymphoid tissue, and skin), corresponding to Grade B, EBM 1b levels. Whether effects seen on the thyroid, liver, spleen, thymus, pituitary, and stomach were due to bias, confounding, or chance, remains an open question.

#### 4.1.3. Carcinogenicity Based on Mechanistic or Other Relevant Studies

A broad discussion of mechanistic research is beyond the scope of this review. Studies investigating genotoxic and clastogenic effects of antimony have been reviewed elsewhere [14] and are briefly mentioned here. Regarding the clastogenic potential of antimony species, recent studies [16,33,43] contradict older [34] evidence of clastogenicity. DNA repair could be impaired through antimony by the inhibition of nucleotid excision repair (NER) through the interaction with a zinc finger domain [48] and by the inhibition of a double stranded break (DSB) repair (a non-homologous end joining (NHEJ) as well as homologous recombination (HR)) [49]. This interference with DNA repair exists in known carcinogens such as nickel and arsenic [50]. Furthermore, antimony may promote mutagenesis through increase in reactive oxygen species (ROS) [51]. DNA damage in lungs of mice was induced in vivo by the inhalation of antimony trioxide, but not in rats [16]. 

### 4.2. Occupational Health Risk Assessment

Given the potential occupational health hazard identified in the previous sections, an occupational health risk assessment is warranted. We will assume that risk = severity x probability x exposure, which is a general model for assessing risk. Based upon predominantly inhalation studies, lung cancer is the most probable adverse effect on which data exist in humans and animals and has a very high severity.

Based on the supportive and supportive/key human studies, lung cancer probability for exposed vs. non-exposed groups was slightly elevated, based on a significant positive trend with an amount of exposure [36] and estimates of enhanced lung cancer mortality of 1.39 [38] and 1.54 [35] (calculated by the author of this study). Based on animal studies, estimates of probability of lung cancer for exposed vs. non-exposed groups were elevated for mice but not for rats. The relative risk for exposed mice was around five, depending on exposure group [16].

Exposure levels from which possible carcinogenic effects arise in humans are impossible to determine form human studies, as human studies have not sufficiently collected individual external or internal exposure data. In animals, 2 years of exposure to at least 3 mg/m^3^ antimony trioxide lead to preneoplastic effects (i.e., epithelium hyperplasia) in lungs (rats and mice) and neoplastic effects (mice) [16]. Occupational exposures in the past have been occasionally reported to attain a similar range, over tenfold (5 mg/m^3^) of todays 0.5 mg/m^3^ of TLV [12,38]. Workers live longer and can be exposed for longer than 2 years. Therefore, a potential for malignant transformation is greater. Compared to decennia ago, exposure levels have however diminished as a result of automation and improved working conditions [12,52].

Consequentially, we can conclude that risk of cancer cannot be excluded in current real-life working conditions, especially in workers with the highest exposures, and thus further scrutiny is warranted. Whether or not this implies a tightening of threshold levels remains an open question based on the available evidence. 

### 4.3. Study Limitations

Firstly, an interpretation of results from, especially, human studies should be viewed within the limitations of these individual articles (as described in detail under results). 

Secondly, publication bias was not examined in detail. As only significant findings were reported in the data extraction and chance findings are possible due to multiple testing in individual (predominantly human toxicology) studies, one should consider findings in this light.

Thirdly, some relevant articles (e.g., from national and regulatory instances) might miss from the final article pool as only two databases were searched (Medline and ToxLine). From the studies listed, it was not possible to determine which internal exposure (BEI) levels would be toxic in humans. Studies examining lung concentrations of antimony failed to see a different concentration in deceased workers compared to controls but lacked sufficient sample sizes [25,37]. 

Lastly, when considering findings of this review, two nuances should be kept in mind, the former concerning sex and the latter concerning study goal. Human studies involved men only. As sex differences in animal studies were observed and background exposure between both sexes may differ, data in men are not to be readily extrapolated to women.

The primary goal (like the IARC Monographs and unlike regulatory substances [53]) of this study was to identify a potential hazard for occupational exposure to antimony species. Only secondarily, and briefly, was the applicability of this risk in real-life working situations addressed. Available research did not permit a broader evaluation. 

### 4.4. Suggestions for Further Research

Future occupational carcinogenicity studies should try to minimize bias and confounding. This can be done through prospective cohort studies with regular screening for cancer, exposure measuring to a wide gamut of substances for individual workers, or speciation studies, minimizing healthy worker effect, large sample sizes, long follow up, and adequate reference populations. More harmonized internal and external exposure data for humans is needed to determine levels from which carcinogenic effects arise. Based on this, TLV’s should be reconsidered.

Regarding animal studies, the relationship between antimony (trioxide) exposure and lymphoma, skin, and adrenal tumors established in the NTP studies deserve further attention. To our knowing, an explanation for observed sex differences in those inhalation studies, especially lymphoma in female mice and skin tumors in male mice is lacking.

Two relatively recent studies [26,27] have established the possibility that antimony acts as an endocrine disruptor through an interaction with estrogen and androgen receptors, promoting tumor growth in the reproductive system. Relevance to carcinogenesis in vivo in rodents and humans is unclear.

The conclusions of our study seem somewhat contradictory to those of NHANES in 2016 [54]. A relationship between inhalation exposure to antimony trioxide and its urinary excretion was established by those authors. However, that study found no relationship between urinary excretion of antimony and lung cancer, possibly because exposure doses in general populations may not attain carcinogenic levels.

One last important point to keep in mind is the possibility that carcinogenic effects of antimony are attenuated, augmented, or only present when concomitant exposure to other substances exist [54] For instance, such an interaction appears to exist between arsenic and antimony [55] or could be the case for selenium and antimony [25].

## 5. Conclusions

The evidence for carcinogenicity of antimony in occupational exposure settings is inadequate. An increased risk of lung cancer is possible. The evidence for carcinogenicity (lung neoplasms) of antimony inhalation as a trioxide in experimental animals is sufficient. Some possible mechanisms have been identified. Overall, this makes carcinogenicity in humans probable (IARC 2A). As of now, however, it is unclear from what occupational exposure duration and dose this effect could arise and whether TLV’s should be reconsidered. Based on past exposure data, further scrutiny is warranted.

## Figures and Tables

**Table 1:** Antimony compounds.

Compound Name	Chemical Formula	CAS-Number
Trivalent Positive (+III)		
antimony trioxide	Sb_2_O_3_	1309-64-4
antimony trisulfide	Sb_2_S_3_	1345-04-6
sodium stibogluconate	C_12_H_20_O_17_Sb_2._3Na9H_2_O	16037-91-5
antimony potassium tartrate	C_8_H_4_K_2_O_12_Sb_2_	28300-74-5
antimony trifluoride	SbF_3_	7783-56-4
antimony triiodide	SbI_3_	7790-44-5
antimony acetate	Sb(CH_3_COO)_3_	6923-52-0
antimony trichloride	SbCl_3_	10025-91-9
Trivalent Negative (−III)		
stibine	SbH_3_	7803-52-3
Elemental (0)		
elemental antimony	Sb	7440-36-0
Pentavalent Positive (+V)		
antimony pentafluoride	SbF_5_	7783-70-2
antimony pentachloride	SbCl_5_	7647-18-9

**Table 2:** Study design.

Review Question: Population, Exposure, Comparison, and Outcome (PECO) (Table 3)					
Literature Review					
Human Toxicology				Animal Toxicology	
Pubmed		ToxNet		Pubmed	ToxNet
Development of search terms		Development of search terms		Development of search terms	Development of search terms
Development of Inclusion/Exclusion criteria				Development of Inclusion/Exclusion criteria	
Independent selection of articles by both reviewers		Independent selection of articles by both reviewers		Independent selection of articles by both reviewers	Independent selection of articles by both reviewers
Solving discrepancies between both reviewers		Solving discrepancies between both reviewers		Solving discrepancies between both reviewers	Solving discrepancies between both reviewers
Articles excluded (no original data)		Articles excluded (no original data)		Articles excluded (no original data)	Articles excluded (no original data)
				Additional search with new search terms	Additional search with new search terms
				Independent selection of articles by both reviewers	Independent selection of articles by both reviewers
				Solving discrepancies between both reviewers	Solving discrepancies between both reviewers
				Articles excluded (no original data)	Articles excluded (no original data)
Eliminating duplicate articles				Eliminating duplicate articles	
Final pool of Human Toxicology studies				Final pool of Animal Toxicology studies	
Data extraction (Table 4)				Data extraction (Table 5)	
Quality and Risk of Bias (ROB) assessment of individual studies (Supplementary Material Table S5)				Quality and Risk of Bias (ROB) assessment of individual studies (Supplementary Material Table S6)	
Human Exposure Evidence Integration and Evidence Level				Animal Exposure Evidence Integration and Evidence Level	
Mechanistic and other relevant elements					
**Occupational health risk assessment**					
**Suggestions for further research**					
**Study limitations**					
**General Evidence Integration and Conclusion**					

**Table 3:** Population, Exposure, Comparison, and Outcome (PECO).

**Population**
Workers or occupationally exposed people, including possibly sensitive subgroups
**Exposure**
Exposure to antimony and antimony compounds (all substances containing antimony), through all possible exposure routes (dermal, inhalation and oral ingestion):
- Elemental antimony;
- Trivalent antimony (III) and species: Antimony trioxide, antimony trisulfide, antimony trifluoride, antimony trichloride, antimony tribromide, antimony triiodide, antimony potassium tartrate,…
- Pentavalent antimony (V) and species: Antimony pentafluoride, antimony pentachloride,…
- Trivalent negative antimony (-III) and species.
**Comparison**
Non-exposure or exposure beneath threshold that produces critical effect
**Outcome**
Development of any type of premalignant lesion, cancer, or malignancy

**Table 4:** Data extraction human studies.

Reference	Industry, Occupation, Exposure	Sample Characteristics	Sample Size (*N*)	Time period	Study Type	Dose of Exposure	Duration of Exposure	Findings
Gerhardsson 1982 [37]	Metal smelting and refinery (Sb species not specified)	Cases: deceased men in northern Sweden, and who died during the period 1976- 1978, divided into 3 groups (I: death caused by malignancy, II: death caused by cardiovascular disease III: death from other causes); Controls: age-matched men from a town 50 km from the factory	Cases: 40 (I: 15, II: 17, III: 8); Controls: 11	Before 1978 (death 1976–1978)	Case-control	Not measured, instead tissue concentrations were measured	Duration of employment, interindividual differences. I: 30,9y +- 7,1; II: 32,5 +- 8,2; III: 28,9 +- 10	Antimony amounts in lung tissue of deceased workers were significantly higher than in the reference group (*p* < 0.001). They did not differ significantly between worker groups.
Sweeney 1985 [22]	Fur workers (Sb species not specified)	Subjects: pensioned fur dressers, compared to Standardized Mortality Ratios (SMR) in the US and in New York City (NYC)	168	Before 1977	Retrospective occupational cohort	Not measured	No data	SMR’s for gross mortality are 118 and 111 (compared to US and NYC rates). Increased risk of death from all malignancies combined (SMR 184 (US) and 151 (NYC), *p* < 0.05), which reflected the significantly higher than expected risk from lung cancer (SMR 232, *p* < 0.05). Mortality from colorectal cancer, as well as from nonmalignant respiratory diseases, was also slightly elevated (not significant).
Wingren 1987 [5]	Glassworks (Sb species not specified)	Subjects: glassworks employees. Referents: rural referents (farmers, forestry workers,…)	Subjects: 887; Referents: 4611	1950–1982	Retrospective occupational cohort	Not measured	Duration of employment	OR for total cancer was 1.3 (90% CI 1.02–1.4), OR for lung cancer 1.9 (90% CI 1.1–2.5), OR for stomach cancer was 1.6 (90% CI 1.1–2.0), OR for colon cancer was 1.7 (90% CI 1.04–2.5). OR for cancer were highest in glassblowers.
Wingren 1990 [23]	Glassworks (Sb species not specified)	Subjects: male art glass workers, compared to expected national and county mortality rates	625	1964–1985	Retrospective occupational cohort	Not measured	At least one month, average exposure per person 15,6 years; 9151 person years at risk	Compared to national and county death rates, a moderate increase in total cancer deaths was observed (not significant; 26 observed cases vs. 22.3 and 18.9 expected from national and county death rates, respectively). Particularly noted for lung cancer, (6 observed cases vs. 4.2 (*p* > 0,1) and 2.5 (*p* < 0,1) expected, respectively), colon cancer (4 observed cases vs. 1.6 expected, not significant), cancer of the pharynx (2 observed cases vs. 0.2 and 0.1 expected, *p* < 0.05 for both), and prostate (4 observed cases vs. 3.0 and 2.4 expected, not significant). A correlation with duration of exposure was seen.
Finkelstein 1991 [39]	Steel manufacturing (Sb species not specified)	Subjects: deceased workers at an electric arc steel making operation, compared to expected provincial mortality rates	335	Deceased before 1989	Retrospective occupational cohort	Below 10% of TLV for antimony	Ever worked	Eight of thirty men who had ever worked in the pouring pit area died of lung cancer (PMR=provincial mortality rate 276; *p* ≤ 0.01); non-significant excess mortality for lung cancer among other melting department employees (PMR 145, *p* = 0.1); excess mortality as well for gallbladder cancer (PMR 1000, *p* = 0.04). There was a significant correlation between lung cancer risk and duration of exposure.
Gerhardsson 1993 [25]	Metal smelting/refinery (antimony sulfides and oxides)	Cases: deceased male smelter workers. Controls: 15 rural and 10 urban controls.	85	Before late 1970’s	Case-control	Not measured	26 to 32 years	An 11-fold higher concentration of Sb in lungs of smelter workers was seen, not associated with higher incidence of lung cancer (compared to non-lung cancer smelter workers or rural or urban controls).
Wingren 1993 [4]	Glassworks (Sb species not specified)	Subjects: glassworks employees. Referents: rural referents (farmers, forestry workers,…)	Subjects: 887; Referents: 4611	1950–1982	Retrospective occupational cohort	Qualitatively stratified (none, low or high), not measured	Duration of employment, interindividual differences	OR (with 90% CI) for colon cancer were correlated with exposure: no exposure to antimony 1.4 (0.6–3.3) low exposure 1.8 (0.8–13.8) high exposure 5.0 (2.6–9.6); no excess risk for lung cancer and a decrease in stomach cancer risk were seen (no significance level reported).
Jones 1994 [35]	Metal smelting/refinery (antimony oxides)	Subjects: antimony workers, compared to expected national and county death rates	525	Appr. 1940–1992 (study enrollment and follow-up period); split into two groups (before and after 1961)	Retrospective occupational cohort	Average levels of air contamination: 0,0005–0,11 mg/m3	At least 3 months	An excess of all neoplasm mortality in antimony workers (69 vs 54.7 expected, *p* < 0.07) was seen, attributable to excess lung cancer mortality (37 vs. 23.9 expected, *p* = 0.016). This excess was not observed for workers recruited after 1/1/1961.
Schnorr 1995 [38]	Antimony smelting (Sb species not specified)	Antimony smelter workers of predominantly hispanic ancestry, compared to national and ethnic specific death rates	1014 (Hispanics: 923; white Texans: 91)	1937–1971 (employment and follow-up period))	Retrospective occupational cohort	Eight hour TWA for Sb exposure in 1975: mean 0,551 mg/m3 (range 0,110–2,0); in 1976 mean 0,747 mg/m3 (range 0,05–6,2)	At least 3 months, 33,773 person years at risk, of which 6.8 years employment per person on average	Overall cancer mortality (SMR 0.88; 95% CI 0.72–1.06) compared to US white mortality rates was diminished. This was expected among the Hispanic subgroup studied. Standardized Mortality Ratios (SMR) for lung cancer were (95% CI): 0.75 (0.51–1.07) and SMR (90% CI) 1.39 (1.01–1.88) relative to U.S. white male mortality rates and Texas ethnic-specific mortality rates, respectively. An increase with duration of employment was seen. A significant excess in mortality was observed for cancers of the liver, biliary tract, and gall bladder (SMR 3.17; 95% CI 1.27–6.52 and SMR 1.58; 95% CI 0.57–3.44 relative to U.S. white male mortality rates and Texas ethnic-specific mortality rates, respectively). Stomach cancer was elevated, but not significantly (SMR 1.49; 95% CI 0.71–2.74 and SMR 1.24; 95% CI 0.50–2.55 relative to U.S. white male mortality rates and Texas ethnic-specific mortality rates, respectively)
Jones 2007 [36]	Metal smelting/refinery (Sb species not specified)	Subjects: 1462 male workers at a former tin smelter compared to expected national death rates	1462	1972–1991, with back-extrapolation of expsoures until 1937	Retrospective occupational cohort	1972–1991: 0,37 mg/m3 (0,001–2,7); extrapolation of exposure data to 3 different scenarios (A, B, C). scenario A: 0,59 (0,003–3,3); scenario B 0,62 (0,003–3,7); scenario C 0,63 (0,003–4,7)	35 942 person-years at risk	Weighted gradients (assuming that the resulting excess relative risk of lung cancer declines with time since exposure and attained age) and unweighted gradients were calculated for the relationship between Antimony exposure and lung cancer risk. None of the unweighted gradients were significant (significance level 0.05), whereas all of the calculated weighted gradients were significant: A, Sb weighted: 1.66 (0.56, 3.77 *p* = 0.004); B Sb weighted: 1.18 (0.28, 3.08 *p* = 0.013); C Sb weighted 1.20 (0.35, 2.09 *p* = 0.016)).

**Table 5:** Data extraction animal studies.

Animal Studies - Inhalation									
Reference	Antimony Compound	Study Animal and Sex (M: Male, F: Female)	Sample Size	Dose of Exposure	Duration of Exposure	Examinations Relevant to Cancer Development Assessment	Principal Findings	LOAEL/NOAEL	Notes
Watt 1983 [21]	Antimony trioxide	CDF rats (only F)	13, 17 and 18 per exposure group, respectively	0, 1.6, 4.2 mg/m^3^	1 year/2 years	Unknown, data extracted from NTP report	Scirrhous lung carcinoma significantly increased in highest exposure group (*p* < 0.01); Squamous cell lung carcinoma not significantly increased; Alveolar/bronchiolar lung adenoma: not significantly increased. Significantly increased potential preneoplastic* lesions in all exposure groups: foci of fibrosis, lungs grossly mottled; pneumocyte hyperplasia; increases in incidence of multinucleated giant cells in exposed groups (no *p* value reported).	LOAEL for potential preneoplastic lung lesions (hyperplasia, fibrosis, grossly mottled lungs): 1.6 mg/m^3^ TWA and LOAEL for lung carcinoma: 4.2 mg/m^3^ TWA	* interpretation in NTP 2017, authors didn’t make statement about preneoplastic lesions; calculations of significance based on NTP 2017
Groth 1986 [41]	Antimony trioxide	Albino Wistar Han rats (M/F)	90 per sex per exposure group	0 and 45 or 46 mg/m^3^ (range 0-91 mg/m^3^)	7 hours/day, 5 days/week during 71–73 weeks with intermediate sacrifice at 6, 9 and 12 months (5 per sex per group)	Full necropsy	No lung neoplasms in male rats. Significant increase in lung neoplasms in exposed female rats: total 19/89 vs. 0/89 (*p* < 0.001); Squamous cell carcinoma 9/89 vs. 0/89 (*p* < 0.01); Scirrhous carcinoma 5/89 vs. 0/89 (*p* < 0.05); Bronchioalveolar Adenoma 11/89vs 0/89 (*p* < 0.001). In females, histopathologic lung changes (not quantified): increase in dense particles aggregates, alveolar wall thickening consisting of interstitial fibrosis and alveolar cell-wall hypertrophy and hyperplasia; sometimes cuboidal and columnar cell metaplasia occurred from these foci. Foci containing cholesterol clefts were observed. Sometimes neoplasms arose from sites of interstitial fibrosis. In males: similar changes were seen, but less alveolar protein, less foci with cholesterol clefts, more metaplasia, more leukocytes, additionally eosinophilic material resembling amyloid No significant differences in other types of cancer.	LOAEL for lung neoplasms in female rats: 45 mg/m^3^ TWA	
	Antimony ore concentrate (principally stibnite, Sb2S3)	Albino Wistar Han rats (M/F)	90 per sex per exposure group	0 and 36 or 40 mg/m^3^ (range 0 -191)	7 hours/day, 5 days/week during 71–73 weeks with intermediate sacrifice at 6, 9 and 12 months (5 per sex per exposure group)	Full necropsy	No lung neoplasms in male rats. Significant increase in lung neoplasms in exposed female rats vs. non-exposed rats: total 17/89 vs. 0/89 (*p* < 0,001); histopathology of the lungs in female and male rats exposed to Sb ore was qualitatively similar to that seen with the Sb2O3-exposed animals. The only differences for males were fewer particles and the presence of some birefringent particles, as well as granulomas in tracheobronchial lymph nodes. No significant differences in other types of cancer.	LOAEL for lung neoplasms in female rats: 36 mg/m^3^ TWA	
Newton 1994 [47]	Antimony trioxide	Fischer 344 rats (M/F)	50 per sex per exposure group	0, 0.25, 1, 5, and 25 mg/m^3^	6 h/day, 5 d/week for 13 weeks followed by 27 week postexposure period	Twice daily observation, gross post mortem examinations, histopathology of heart and airways	Survival equal. Elevated absolute and relative lung weights in two highest exposure groups. Leukemia (typical finding at this age in this species). Increase in lung lesions related to exposure: chronic interstitial inflammation, interstitial fibrosis, granulomatous inflammation in highest exposure groups, bronchiolar/alveolar hyperplasia, increase in alveolar macrophages (no calculation of significance).	NOAEL for carcinoma 25 mg/m^3^	
	Antimony trioxide	Fischer 344 rats (M/F)	65 per sex per exposure group	0, 0.05, 0.5, and 5 mg/m^3^	6 h/day, 5 d/week for 52 weeks followed by 52 week postexposure period	Twice daily observation, gross post mortem examinations, histopathology of heart and airways	Increase in potential* preneoplastic lung lesions related to exposure: chronic interstitial inflammation in highest exposure group (*p* < 0.001), interstitial fibrosis (unclear if significant and at what exposure level), granulomatous inflammation (unclear if significant and at what exposure level), bronchiolar/alveolar hyperplasia (unclear if significant and at what exposure level), increase in alveolar/intra-alveolar/peribronchial macrophages (significant from 0.05 mg/m^3^).	NOAEL for carcinoma: 5 mg/m^3^; LOAEL for potential* preneoplastic lung lesions (increase in macrophages): 0.05 mg/m^3^	* interpretation in NTP 2017, authors didn’t make statement about preneoplastic lesions; calculations of significance based on NTP 2017
NTIS/NTP 2016 [16]	Antimony trioxide	Wistar Han Rats (M)	50 per sex per exposure group	0,3,10, 30 mg/m^3^	two years, 5 d per week, 6 h per day, with interim sacrifice at 6 and 12 months	Full necropsy	In lung tissue: Alveolar/bronchiolar adenoma or carcinoma: no significant difference; Alveolar/bronchiolar carcinoma: no significant difference; Alveolar/bronchiolar adenoma no significant difference. Significantly increased preneoplastic lesions (*p* < 0.01 for all exposed groups): lung alveolar epithelium hyperplasia; lung bronchiole epithelium hyperplasia. In adrenal gland: benign pheochromocytoma and adrenal medulla hyperplasia significantly increased in highest exposure group (both *p* < 0.05).	LOAEL for potential preneoplastic lung lesions (alveolar/bronchiolar epithelium hyperplasia): 3 mg/m^3^ exposure dose; NOAEL and LOAEL for adrenal medulla 10 and 30 mg/m^3^	
		Wistar Han Rats (F)	50 per exposure group	0,3,10, 30 mg/m^3^	two years, 5 d per week, 6 hper day, with interim sacrifice at 6 and 12 months	Full necropsy	In lung tissue: Alveolar/bronchiolar adenoma or carcinoma: no significant difference; Alveolar/bronchiolar carcinoma: no significant difference; Alveolar/bronchiolar adenoma: significant increase in two highest exposure groups (*p* < 0.05). Significantly increased preneoplastic lesions: lung alveolar and brochiole epithelium hyperplasia (*p* < 0.01 for all exposed groups); In adrenal gland: benign and malignant pheochromocytoma (both *p* < 0.01) and medullar hyperplasia (*p* < 0.05) significantly increased in highest exposure group.	LOAEL for potential preneoplastic lung lesions (alveolar/bronchiolar epithelium hyperplasia): 3 mg/m^3^ exposure dose; NOAEL and LOAEL for adrenal medulla 10 and 30 mg/m^3^	
		Wistar Han Rats (M/F)	5 per sex exposure group	0, 3.75, 7.5, 15, 30, or 60 mg/m^3^	6 h plus T90 (12 minutes) per day, 5 d per week for 12 exposure days during a 16-day period	Full necropsy	Lung weights in 30 and 60 mg exposure group increased (*p* < 0.05). Incidences of chronic inflammation (increased numbers of alveolar macrophages, perivascular infiltrates of lymphocytes, monocytes, and neutrophils) were significantly increased in 30 and 60 mg exposure groups (*p* < 0.01). In areas of more intense inflammation, the alveolar architecture was sometimes obscured by inflammatory cells, cell debris, and fibrin, and was accompanied by Type 2 alveolar epithelial cell hyperplasia. No gross observations associated with exposure to antimony trioxide noted at necropsy.	NOAEL is 15 mg/m^3^ and LOAEL is 30 mg/m^3^ for chronic inflammation with hyperplasia and NOAEL and LOAEL for increased lung weights: 15 and 30 mg/m^3^	
		Wistar Han Rats (M/F)	5 per sex per exposure group	0,3,10, 30 mg/m^3^	one year, 5 d per week, 6 h per day	MNPCE, MNNCE, PCE, and DNA Comet Assay	No increases in MNPCE, MNNCE, PCE or DNA damage in lung tissue samples or blood leukocytes were observed in male or female Wistar Han rats following exposure to antimony trioxide for 12 months.	NOAEL for MNPCE, PCE and DNA damage in lung tissue: 30 mg/m^3^	MNNCE: micronucleated normochromatic erythrocytes; PCE: immature reticulocytes: sign of bone marrow toxicity
		B6C3F1/N mice (M)	50 per exposure group	0,3,10, 30 mg/m^3^	two years, 5 d per week, 6 h per day, with interim sacrifice at 6 and 12 months	Full necropsy	In lung tissue: Alveolar/bronchiolar adenoma and Alveolar/bronchiolar carcinoma significantly increased among all exposure groups (*p* < 0.001). Significantly increased preneoplastic lesions: lung lymphocyte infiltration and alveolar epithelium hyperplasia (both *p* < 0.001, for all exposed groups).	LOAEL for lung carcinoma and preneoplastic lesions: 3 mg/m^3^	
		B6C3F1/N mice (F)	50 per exposure group	0,3,10, 30 mg/m^3^	two years, 5 d per week, 6 h per day, with interim sacrifice at 6 and 12 months	Full necropsy	In lung tissue: Alveolar/bronchiolar adenoma and Alveolar/bronchiolar carcinoma significantly increased among all exposure groups (*p* < 0.01). Significantly increased preneoplastic lesions: lung lymphocyte infiltration (*p* <0.001 for all exposed groups) and alveolar/bronchiolar epithelium hyperplasia (both *p* < 0.01 for all exposed groups). Malignant lymphoma in whole body significantly increased (*p* < 0.05 for low exposure and *p* <0.001 for higher exposure);	LOAEL for lung carcinoma, preneoplastic lung lesions and malignant lymphoma: 3 mg/m^3^	
		B6C3F1/N mice (M/F)	5 per sex per exposure group	0, 3.75, 7.5, 15, 30, or 60 mg/m^3^	6 h plus T90 (12 minutes) per day, 5 d per week for 12 exposure days during a 16-day period.	Full necropsy	Relative lung weights were significantly increased in 60 mg/m^3^ males (*p* < 0.01) and in 15 mg/m^3^ females (*p* < 0.01); absolute and relative lung weights already were increased at 7.5 mg/m^3^ for males and females, respectively. In the larynx, there were significantly increased incidences of squamous metaplasia of the epiglottis from 30 mg/m^3^ on. No gross observations associated with exposure to antimony trioxide noted at necropsy.	LOAEL for increased absolute (M) and relative (F) lung weights; NOAEL is 3.75 mg/m^3^	
		B6C3F1/N mice (M/F)	5 per sex per exposure group	0,3,10, 30 mg/m3	one year, 5 days per week, 6 hours per day	MNPCE, MNNCE, PCE, and DNA Comet Assay	Significant increases in MNNCE at highest exposure concentration (*p* < 0.001), and PCE in male (at 10 mg/m^3^, *p* < 0.001)) and female (at 30 mg/m^3^, *p* <0.01) mice. Increased levels of DNA damage in male 3 mg/m3 and female highest exposure group (both *p* <0.001) lung tissue samples, but not in peripheral blood leukocytes.	LOAEL (lung DNA damage): 3 mg/m^3^	
**Animal Studies - Ingestion**									
Reference	Antimony Compound	Study animal and sex (M: male, F: female)	Sample size	Dose of Exposure	Duration of exposure	Examinations relevant to cancer development assessment	Principal findings	LOAEL/NOAEL	Notes
Kanisawa 1969 [44]	Antimony potassium tartrate (APT)	Swiss albino mice (M/F) of the Charles River Strain (CD-1)	76 for Antimony exposure and 71 controls	5 mcg/ml drinking water	Lifetime**	Sections of five tissues and gross tumors	Compared to controls, significant differences in the incidences of spontaneous tumors and malignant tumors did not appear.	NOAEL 5 mcg/mL drinking water	**(50% dead males: 582 days, 90% dead males: 651; 50% dead females: 576 days, 90% dead females: 742 d)
Schroeder 1970 [45]	Antimony potassium tartrate (APT)	Long-Evans strain rats (M/F)	51 male and 59 female rats (antimony group) and 52 male and 54 female rats (control group)	5 ppm in drinking water	Lifetime**	Survival time and development of grossly visible tumors	Survival time reduced in antimony fed rats (on average 106 d less for male and 107 d less for females). No significant difference in macroscopic tumor development	LOAEL for increased mortality: 5 ppm drinking water	** mean per sex: M 746 days (90% dead: 987 days) and F 797 days (90% dead 992 d)
Ainsworth 1991 [42]	Antimony trioxide	Microtus agrestis (short tailed field vole)	According to exposure duration: 8, 10, 8, 8 and 12, 8	500 mg Sb/kg food;	30, 40, 50, 60 d and 30+5 recovery, 30+10 recovery	Not explicitly stated	No obvious harmful effects: The appearance of the internal organs of voles receiving the 500 mg Sb/ kg diet was indistinguishable from that of the control voles. Wet and dry weights of the liver, kidney, and lungs of animals receiving the 500 mg Sb /kg diet were not significantly different from those of control animals.	NOAEL 500 mg/kg food dose	
	Antimony trioxide	Microtus agrestis (short tailed field vole)	6 per exposure group p	20000 mg Sb/kg food	12 d	Not explicitly stated	Animals remained healthy. No histological changes compared to control group voles could be observed in the sections of liver and kidney examined by electron microscopy.	NOAEL 20,000 mg/kg food exposure monodose	
Gurnani 1992 (1)[32]	Antimony trioxide	Swiss albino mice (M/F)	5 per exposure group	0, 400, 666.67 and 1000 mg/kg body mass	6, 12, 24 h	Chromosomal abberations: chromatid gaps, chromatid breaks, centric fusions and polyploidy in bone marrow cells	In the acute exposure groups, no clastogenic effects were seen.	NOAEL 1000 mg/kg body weight dose	
	Antimony trioxide	Swiss albino mice (only M)	5 per exposure group	0, 400, 666.67 and 1000 mg/kg body mass	7, 14 and 21 d	Chromosomal abberations: chromatid gaps, chromatid breaks, centric fusions and polyploidy in bone marrow cells	In the 21d exposure groups, dose dependent chromosomal abberations were seen for both sexes (*p* < 0.001 for trend). The highest dose was lethal on day 20 of treatment. No relationship was seen with duration of exposure.	LOAEL for CA: 400 mg/kg body weight	
Gurnani 1992 (2)[20]	Antimony trichloride	Swiss albino mice	Unknown	Unknown, several	6, 12, 24 h	Chromosomal abberations in bone marrow cells	Frequencies of chromosomal aberrations (DNA strand breaks principally) were directly related to the dose used and were significantly higher than the control.***	Inference not possible based on abstract	*** data extracted from abstract
Dieter 1992 [31]	Antimony potassium tartrate (APT)	Fischer 344 rats (M/F)	5 per sex for each dose group	0, 0.15, 0.30, 0.65, 1.25, and 2.5 mg/mL in drinking water or cumulative daily dose of 0, 16, 28, 59, 94, or 168 mg/kg	14 d	Clinical signs, clinical pathology of several tissues, hematology tests	No clinical nor histopatholigical signs of APT Toxicity in none of the exposure groups	NOAEL of 168 mg APT/kg body weight	
	Antimony potassium tartrate (APT)	B6C3F1 mice (M/F)	5 per sex for each dose group	0, 0.30, 0.65, 1.25, 2.5, and 5.0 mg/mL or cumulative daily dose of 0, 59, 98, 174, 273, or 407 mg/kg	14 d	Clinical signs, clinical pathology of several tissues, hematology tests	Effects were only seen in 407 mg/kg group: Clinical signs of toxicity: impaired weight gain, rough haircoat, emaciation, abnormal posture, hypoactivity, and decreased fecal material, consistent with avoidance of the APT-dosed water. Histopathological signs: in forestomach: necrosis, ulceration, inflammation and focal hyperplasia of squamous epithelium; in hepatocytes: centriblobular cytoplasmic vacuolization.	NOAEL of 273 mg APT/kg body weight, LOAEL for stomach squamaous epithelium hyperplasia: 407 mg APT/kg body weight	
Poon 1998 [46]	Antimony potassium tartrate (APT)	Sprague–Dawley Crl:CD (SD) rats (M/F)	95 male and 95 female, divided in groups	groups: control (25), 0.5 ppm (15), 5 ppm (15), 50 ppm (15) and 500 ppm (25)	90 d	Full Necropsy	No clinical signs of toxicity; A male rat in the highest dose group had a cirrhotic liver and a female rat in the lowest dose group had a nodular, fibrotic, spleen. Persistent dose-dependent histological changes in thyroid gland, perisistent dose dependent histological changes in liver (irreversible after 4 wk recovery period), very mild treatment-related changes in spleen developing predominantly in recovery period; changes in thymus in recovery period; minimal non-dose dependent changes in pituitary gland.	inference not possible based on paper	
Elliott 1998 [43]	Antimony trioxide	CD-1 mice (M/F)	Not indicated	5000 mg/kg Antimony Trioxide (single dose) OR 400, 666.67, 1000 mL/kg/day	single dose or 7, 14 and 21 d	Micronucleated Polychromatic Erythrocytes (MNPCE)	No difference in micronucleated polychromatic erythrocytes in both species; significant decrease in polychromatic erythrocytes in female mice for single dose study	NOAEL for MNPCE: 5000 mg/kg monodose or 1000 ml/kg repeated dose LOAEL for decrease in PCE: 5000 mg/kg in single dose	
	Antimony trioxide	Alderley Park Alpk: APfSD rats (only M)	Not indicated; per animal 2000 polychromatic erythrocytes and 1000 erythrocytes	3200 or 5000 mg/kg	Single dose	Liver DNA repair assay	No significant differences	NOAEL for liver DNA repair: 1000 ml/kg	
Kirkland 2007 [34]	Antimony trioxide	Sprague–Dawley Crl:CD (SD) rats (M/F)	6 per exposure group per sex	1000 mg once and 250 or 500 or 1000 mg/kg food/day Sb2O3 and positive control CycloPhosphAmid 20 mg/kg once or vehicle controls	Single dose or 21 d	Micronuclei (MN) and chromosomal abberations (CA)	No clinical signs of toxicity. No difference in mitotic indexes between exposed groups and unexposed groups; chromosomal abberations in Sb2O3 treated groups were low (falling within normal ranges) and not significantly higher than in vehicle controls; Micronucleated polychromatic erythrocytes (MNPCE): % PCE not reduced in Sb2O3 treated groups as compared to vehicle control; MN frequencies in Sb2O3 exposed groups within normal ranges and not significantly different from vehicle control groups	NOAEL for MNPCE and CA: 1000 mg/kg food dose	
**Animal Studies - Dermal contact**									
Reference	Antimony Compound	Study animal and sex (M: male, F: female)	Sample size	Dose of Exposure	Duration of exposure	Examinations relevant to cancer development assessment	Principal findings	LOAEL/NOAEL	
NTIS/NTP 2016 [16]	Antimony trioxide	B6C3F1/N mice (M/F)	50 per sex per exposure group	0,3,10, 30 mg/m^3^	Two years, 5 d per week, 6 hper day, with interim sacrifice at 6 and 12 months	Skin histopathology	Significant increase in benign fibrous histiocytoma and fibrous histiocytoma/fibrosarcoma (*p* < 0.05 for both in highest exposure groups) in males; non significant increase in squamous cell metaplasia in females	LOAEL for male mice and NOAEL for female mice: 30 mg/m^3^	

## Supplementary Materials

The following are available online at https://www.mdpi.com/1660-4601/16/22/4474/s1. Table S1: Search string development, Table S2: Inclusion/exclusion criteria, Table S3: Study selection human toxicology studies, Table S4: Study selection animal toxicology studies, Table S5: Critical appraisal human toxicology studies, Table S6: Critical appraisal animal toxicology studies.
