# Supplementary material for: Risk of Cancer for Workers Exposed to Antimony Compounds: A Systematic Review
Source: Int J Environ Res Public Health. 2019 Nov 14;16(22):4474. doi: 10.3390/ijerph16224474 (PMC6888331; doi:10.3390/ijerph16224474)
Supplement: Supplementary file 1 [file ijerph-16-04474-s001.pdf]

Supplementary Table S1. Search string development.

**Human Toxicology**
In PubMed
("Antimony"[Mesh] OR "antimony")
hits: 6960
("Occupational Exposure"[Mesh] OR "work *") AND ("Antimony"[Mesh] OR "antimony")
hits: 328
("Occupational Exposure"[Mesh] OR "work *"[All Fields]) AND ("Antimony"[Mesh] OR "antimony"[All Fields]) AND ("Neoplasms"[Mesh] OR "cancer"[All Fields] OR "canc*"[All Fields])
hits: 26 (28/06/2018)
In ToxLine
(occupational exposure OR work *) AND antimony AND (cancer OR neoplas *)
hits: 113 (28/06/2018)
**Animal and Mechanistic Toxicology**
In PubMed
("Mammals"[Mesh] AND ("Antimony"[Mesh] OR "antimony"[All Fields]) AND ("Neoplasms"[Mesh] OR "cancer"[All Fields] OR "canc *"[All Fields]))
hits: 330
("Mammals"[Mesh] AND ("Antimony"[Mesh] OR "antimony"[All Fields]) AND ("Neoplasms"[Mesh] OR "cancer"[All Fields] OR "canc *"[All Fields]) NOT "sentinel")
hits: 261
("Mammals"[Mesh] AND ("Antimony"[Mesh] OR "antimony"[All Fields]) AND ("Neoplasms"[Mesh] OR "cancer"[All Fields] OR "canc *"[All Fields]) NOT ("sentinel" OR Leishman * OR schistosom *))
hits: 205
("Mammals"[Mesh] AND ("Antimony"[Mesh] OR "antimony"[All Fields]) AND ("Neoplasms"[Mesh] OR "cancer"[All Fields] OR "canc *"[All Fields]) NOT ("sentinel" OR Leishman * OR schistosom *))
hits: 205
("Antimony"[Mesh] OR "antimony"[All Fields]) AND ("Neoplas *" OR "cancer"[All Fields] OR "carcinog *"[All Fields]) NOT ("sentinel" OR Leishman * OR schistosom *)
hits: 117 (28/06/2018)
Additional PubMed search
("antimony"[MeSH Terms] OR "antimony"[All Fields]) AND ("toxicity"[Subheading] OR "toxicity"[All Fields]) AND ("In Vivo"[Journal] OR "In Vivo (Brooklyn)"[Journal] OR ("in"[All Fields] AND "vivo"[All Fields]) OR "in vivo"[All Fields])
hits: 45 (28/06/2018)
In ToxLine
Mammals AND Antimony AND (Neoplasms OR cancer OR canc *)
hits: 136
antimony AND (Neoplas * OR cancer OR carcinog *)
hits (03/07/2018): 392
Additional ToxLine search
antimony AND toxicity AND "In Vivo"
hits: 31 (03/07/2018)

Supplementary Table S2. Inclusion/Exclusion criteria.

	**Human Toxicology**	
	**Exclusion**	Inclusion
General	complete study unavailable review-article, language other than English, focused on poisoning/overdose, study doesn't contain original data, antimony not mentioned in title/abstract	complete study available, articles written in English, study contains original data, antimony mentioned in title/abstract
Specific	study on environmental/residential everyday exposure to antimony compounds, study in children, study on antimony compounds as treatment of disease (e.g., schistosomiasis, leishmaniasis), no cancer related endpoint, case studies	study on occupational exposure to antimony compounds
	**Animal Toxicology**	
	Exclusion	Inclusion
General	complete study unavailable, review-article, language other than English, focused on poisoning/overdose, study doesn't contain original data, antimony not mentioned in title/abstract	complete study available, articles written in English, study contains original data
Specific	non-mammalian models, endpoint not relevant to PECO or not mentioned, route of administration is other than that of interest (e.g., IV or IM injection), route of administration not mentioned, effects in non-relevant tissues/cells, dose/concentration of exposure is not biologically relevant, dose/concentration of exposure is not relevant/convertible to Reference Value (ReV) development, research concerning drugs and drug development, research concerning antimony compounds in relation to cancer cells, research on scintigraphy or sentinel node procedures	mammalian models, endpoint mentioned and relevant to PECO, route of administration is mentioned and same than that of interest, effects in relevant tissues/cells, dose/concentration of exposure is relevant/convertible to Reference Value (ReV) development

Supplementary Table S3. Study selection human toxicology studies**.**

	**Human toxicology**	
	Pubmed	ToxLine
Search Terms	("Occupational Exposure"[Mesh] OR "work*"[All Fields]) AND ("Antimony"[Mesh] OR "antimony"[All Fields]) AND ("Neoplasms"[Mesh] OR "cancer"[All Fields] OR "canc*"[All Fields])	(occupational exposure OR work*) AND antimony AND (cancer OR neoplas*)
Hits: date and number	28/06/2018: 26	28/06/2018: 113
Articles, excluding duplicates in search, retained independently per reviewer (AS: Anton Saerens; MG: Manosij Ghosh)	AS 7 articles, MG 7 articles	AS 9 articles, MG 11 articles
Articles about which reviewers agreed	7 articles	9 articles
Articles about which reviewers disagreed	0 articles	2 articles: Sweeney 1985[22] Wingren 1990[23]
Additionally included and excluded articles after discussion (+rationale)	0 articles	Articles included after discussion: 2 articles: Sweeney 1985 (Antimony not in Abstract, but included based on full text); Wingren 1990 (Antimony not in Abstract, but included based on full text)
Articles excluded (no original data)	0 articles	1 article: Gerhardsson 1988[24]: uses same data as Gerhardsson 1993[25]
remaining hits:	7	10
	Final pool: 10 articles	

Supplementary Table S4. Study selection animal toxicology studies

	**Animal Toxicology**	
	Pubmed	ToxLine
Search Terms	("Antimony"[Mesh] OR "antimony"[All Fields]) AND ("Neoplas*" OR "cancer"[All Fields] OR "carcinog*"[All Fields]) NOT ("sentinel" OR leishman* OR schistosom*)	antimony AND (Neoplas* OR cancer OR carcinog*")
Hits: date and number	03/07/2018: 117	03/07/2018: 392
Articles, excluding duplicates in search, retained independently per reviewer (AS: Anton Saerens; MG: Manosij Ghosh)	AS 3 articles, MG 5 articles	AS 10 articles, MG 13 articles
Articles about which reviewers agreed	2 articles	10 articles
Articles about which reviewers disagreed	4 articles: Zhang 2018[26] Kotsopoulos 2012[27] Rossi 1987[28] Sunderman 1983[29]	3 articles: Zhang 2018 Sunderman 1983, Rossi 1987
Additionally included and excluded articles after discussion (+rationale)	Articles excluded after discussion: Zhang 2018 (human cancer cells implanted in immunodeficient mice) Kotsopoulos 2012 (no animal study), Rossi 1987 (uncertain relevance to health endpoint), Sunderman 1983 (intramuscular administration)	Articles excluded after discussion: Zhang 2018 (excluded earlier) Sunderman 1983 (intramuscular administration); Rossi 1987 (uncertain relevance to health endpoint)
Articles excluded (no original data)	0 articles	1 article: Dieter 1991[30]: uses same data as Dieter 1992[31]
remaining hits:	2	9
		NB: many of the references identified in this search are duplicates OR are integral parts of other studies identified with this search. In this last case, only the complete studies were counted.

**Animal Toxicology**		
	Pubmed	ToxLine
Additional Search Terms	("antimony"[MeSH Terms] OR "antimony"[All Fields]) AND ("toxicity"[Subheading] OR "toxicity"[All Fields]) AND ("In Vivo"[Journal] OR "In Vivo (Brooklyn)"[Journal] OR ("in"[All Fields] AND "vivo"[All Fields]) OR "in vivo"[All Fields])	antimony AND toxicity AND "*In Vivo*"
Hits: date and number	03/07/2018: 45	03/07/2018: 31
Articles, excluding duplicates in search, retained independently per reviewer (AS: Anton Saerens; MG: Manosij Ghosh)	AS 5 articles, MG 6 articles	AS 7 articles, MG 8 articles
Articles about which reviewers agreed	5 articles	7 articles
Articles about which reviewers disagreed	1 article: Zhang 2018: excluded earlier	1 article: Zhang 2018: excluded earlier
Articles excluded (no original data)	1 article: Gurnani 1993[33]: uses same data as Gurnani 1992[32]	0 articles
remaining hits:	4	7
	Final pool: 13 articles	

Supplementary Table S5. Critical appraisal human toxicology studies

	General study scoring criteria													
Reference	Original data	Applicable route of exposure	Single route	Range of doses/ exposures	Exposure concentration known/measured	Blinded study	Health effects relevant to ReV development	Single chemical exposure	Appropriate endpoints measured	Measured outcomes reported	Study design sufficient/ clearly defined	Calculation of sample size	Confounding factors	Appropriate research practices
Gerhardsson 1982[37]	1	1	0	-1	-1	0/not applicable	1	1	1	1	1	-1	-1	1
Sweeney 1985[22]	1	1	0	-1	-1	0/not applicable	1	-1	1	1	-1	-1	-1	1
Wingren 1987[5]	1	1	0	-1	-1	0/not applicable	1	-1	1	1	1	0	-1	-1
Wingren 1990[23]	1	1	0	-1	-1	0/not applicable	1	-1	1	1	-1	0	-1	1
Finkelstein 1991[39]	1	1	0	-1	1	0/not applicable	1	-1	1	1	-1	0	-1	1
Gerhardsson 1993[25]	1	1	0	-1	-1	0/not applicable	1	-1	1	1	1	-1	-1	1
Wingren 1993[4]	1	1	0	-1	0	0/not applicable	1	-1	1	1	1	0	-1	-1
Jones 1994[35]	1	1	0	-1	-1	0/not applicable	1	-1	1	1	1	0	-1	1
Schnorr 1995[38]	1	1	0	1	1	0/not applicable	1	-1	1	1	1	0	-1	1
Jones 2007[36]	1	1	0	1	1	0/not applicable	1	-1	1	1	1	0	-1	1

	Human study scoring criteria				Study Selection		
Reference	Appropriate comparison groups	Follow up of subjects	Temporal relation	Consistency of results with other available evidence	Total points (General + Human study scoring)	Acute (A) or Chronic (C)	Key (K), Supporting (S), or Informative (I)
Gerhardsson 1982[37]	0	0	-1	0	3	C	I
Sweeney 1985[22]	0	0	1	1	2	C	I
Wingren 1987[5]	0	0	1	0	2	C	I
Wingren 1990[23]	0	0	1	0	2	C	I
Finkelstein 1991[39]	1	0	1	1	6	C	I
Gerhardsson 1993[25]	0	0	-1	0	1	C	I
Wingren 1993[4]	0	0	1	0	3	C	I
Jones 1994[35]	0	1	1	1	6	C	S
Schnorr 1995[38]	0	0	1	1	9	C	S/K
Jones 2007[36]	1	0	1	1	10	C	S

Supplementary Table S6. Critical appraisal animal toxicology studies

	General study scoring criteria													
Reference	Original data	Applicable route of exposure	Single route	Range of doses/ exposures	Exposure concentration known/measured	Blinded study	Health effects relevant to ReV development	Single chemical exposure	Appropriate endpoints measured	Measured outcomes reported	Study design sufficient/ clearly defined	Calculation of sample size	Confounding factors	Appropriate research practices
Schroeder/Kanisawa 1969[44]	1	1	1	0	0	0	1	1	-1	1	-1	-1	0	-1
Schroeder 1970[45]	1	1	1	0	0	0	1	1	-1	1	1	-1	0	1
Watt 1983[21]	scoring impossible based on data of NTP report													
Groth 198641]	1	1	1	0	1	0	1	-1	1	1	1	-1	-1	1
Ainsworth 1991[42]	1	1	1	1	1	0	1	1	-1	0	-1	-1	0	-1
Gurnani 1992 (1)[32]	1	1	1	1	0	0	0	-1	1	1	1	-1	-1	-1
Gurnani 1992 (2)[20]	scoring impossible based on abstract													
Poon 1998[46]	1	1	1	1	0	0	1	1	1	0	0	-1	0	1
Newton 1994	1	1	1	1	1	0	1	1	1	0	1	-1	0	1
Elliott 1998[43] (liver DNA repair and bone marrow micronucleus assay)	1	1	1	1	1	1	0	1	1	1	0	-1	0	1
Dieter 1992[31]	1	1	1	1	1	0	1	1	1	0	1	-1	0	1
Kirkland 2007[34]	1	1	1	1	1	1	0	1	1	1	1	-1	0	1
NTIS/NTP 2016[16]	1	1	1	1	1	1	1	1	1	1	1	-1	0	1

	Animal study scoring criteria						Study Selection		
Reference	Multiple species	Both sexes	Exposure regimes	Study design sufficient/clearly defined	Identical conditions across study groups	Dose applicable to ReV development	Total points (General + Animal study scoring)	Acute (A) or Chronic (C)	Key (K), Supporting (S), or Informative (I)
Schroeder/Kanisawa 1969[44]	0	1	0	1	1	0	5	C	I
Schroeder 1970[45]	0	1	0	1	1	0	9	C	I
Watt 1983[21]	scoring impossible based on data of NTP report						/	C	I
Groth 198641]	0	1	0	1	0	1	9	C	I
Ainsworth 1991[42]	0	0	1	-1	1	0	4	SA	I
Gurnani 1992 (1)[32]	0	0	1	1	1	0	6	A	I
Gurnani 1992 (2)[20]							/	A	I
Poon 1998[46]	0	1	1	1	1	1	12	SA	S
Newton 1994	0	1	1	1	1	1	14	C	S
Elliott 1998[43] (liver DNA repair and bone marrow micronucleus assay)	0	0	1	1	1	0	12	A/SA	I
Dieter 1992[31]	1	1	1	1	1	1	15	A/SA	I
Kirkland 2007[34]	0	1	1	1	1	0	14	SA	I
NTIS/NTP 2016[16]	1	1	1	1	1	1	17	SA/C	K
